# Gaze patterns reflect the retrieval and selection of memories in a context-dependent object location retrieval task

**DOI:** 10.1038/s41598-024-59815-9

**Published:** 2024-04-24

**Authors:** Somang Paeng, Hyoung F. Kim

**Affiliations:** School of Biological Sciences, Seoul National University (SNU), Gwanak-ro, Gwanak-gu, Seoul, 08826 Republic of Korea

**Keywords:** Cognitive neuroscience, Learning and memory

## Abstract

Selective retrieval of context-relevant memories is critical for animal survival. A behavioral index that captures its dynamic nature in real time is necessary to investigate this retrieval process. Here, we found a bias in eye gaze towards the locations previously associated with individual objects during retrieval. Participants learned two locations associated with each visual object and recalled one of them indicated by a contextual cue in the following days. Before the contextual cue presentation, participants often gazed at both locations associated with the given object on the background screen (look-at-both), and the frequency of look-at-both gaze pattern increased as learning progressed. Following the cue presentation, their gaze shifted toward the context-appropriate location. Interestingly, participants showed a higher accuracy of memory retrieval in trials where they gazed at both object-associated locations, implying functional advantage of the look-at-both gaze patterns. Our findings indicate that naturalistic eye movements reflect the dynamic process of memory retrieval and selection, highlighting the potential of eye gaze as an indicator for studying these cognitive processes.

## Introduction

The retrieval and selection of long-term memories are crucial cognitive processes in our daily lives, given the vast number of memories that individuals accumulate. However, contextual cues may be insufficient or delayed, requiring individuals to prepare their behaviors for upcoming situations. One mechanism for this process is retrieving multiple memories associated with a cue, followed by selecting the most relevant target memory when the context is given. Alternatively, the memory retrieval process may only be initiated once the cue becomes sufficient to represent the context.

The memory retrieval and selection process has been studied using the retrieval-induced forgetting paradigm (RIF)^1–4^. The RIF paradigm utilizes the phenomenon where the repeated recall of one memory results in the impairment of the explicit report of related competitor memories later^5,6^. However, the explicit report of memory may not fully reflect this dynamic process because the report itself is the outcome of the memory selection process. A behavioral index that can reflect this process promptly at each step is necessary to better understand the transitory process of memory retrieval and selection. In this context, accessing the internal cognitive process that precedes the resulting explicit response becomes crucial, emphasizing the significance of a behavioral index that can be acquired while minimizing explicit responses.

One plausible approach is utilizing eye movements as a behavioral index for accessing the internal memory retrieval and selection process. Previous studies have indicated a correlation between eye movements and memory retrieval^7–10^. One specific gaze behavior related to memory retrieval is “looking-at-nothing” (LAN) on a blank screen^11–13^. In this behavior, participants directed their gaze towards vacant locations where visual or verbal information was previously encoded while trying to recall previously encoded information. Moreover, eye movements can easily be decoupled from explicit response modalities by task design, and studies have even suggested that memory effects on gaze behavior precede an explicit response^9,14^.

In the context of retrieval and selection among multiple memories, it is known that aligning gaze positions with the encoding location increases successful retrieval of the target memory from multiple associated memories, whereas gazing at locations associated with competing memories leads to increased subsequent forgetting of those competing memories^15^. Additionally, when gazing toward the competitor location that is subsequently forgotten, there was a significantly greater difference between the pupil sizes during repeated retrieval of the target memory.

Building on the insights from these studies, we suggest the use of eye gaze as a tool to investigate which associated memory is currently being retrieved and to reveal the multi-memory retrieval and selection process. To utilize eye gaze as behavioral index to gain insights into multi-memory retrieval and selection, it is crucial to understand naturalistic gaze behaviors relating to these cognitive processes. Thus, we aimed to probe into what spontaneous and voluntary gaze patterns arise when there are multiple memories associated with a given visual object cue, and the context indicating which is the relevant target memory is presented after a delay. Furthermore, our study tracked the dynamics of these gaze patterns over a long-term period of learning and retrieval, further expanding upon the current literature of multi-memory retrieval and selection and the corresponding gaze behaviors.

Specifically, to investigate the multi-memory retrieval and selection process using eye movements, we tracked participants’ free eye movements while they selectively retrieved one of the two locations associated with individual visual objects using joystick manipulation. In our experiment, participants learned the associations between multiple spatial locations and visual objects over five days (day 1–5), and their ability to selectively retrieve the locations was assessed on the next day or three days after learning (day 2–5, day 8). We found that there are prominent patterns of gaze locations that arise spontaneously in the retrieval of multiple memories, and the selection of the context-relevant target memory. During the retrieval phase, participants exhibited gazes toward both locations previously associated with the visual object. Their eye movements converged toward the context-appropriate target location after the contextual cue was presented. Interestingly, these gazes toward both locations associated with the object reflecting multi-memory retrieval correlated with a higher memory retrieval accuracy during certain phases of learning.

## Results

### Participants learned and retrieved memories of the locations associated with individual visual objects

**Figure 1:**
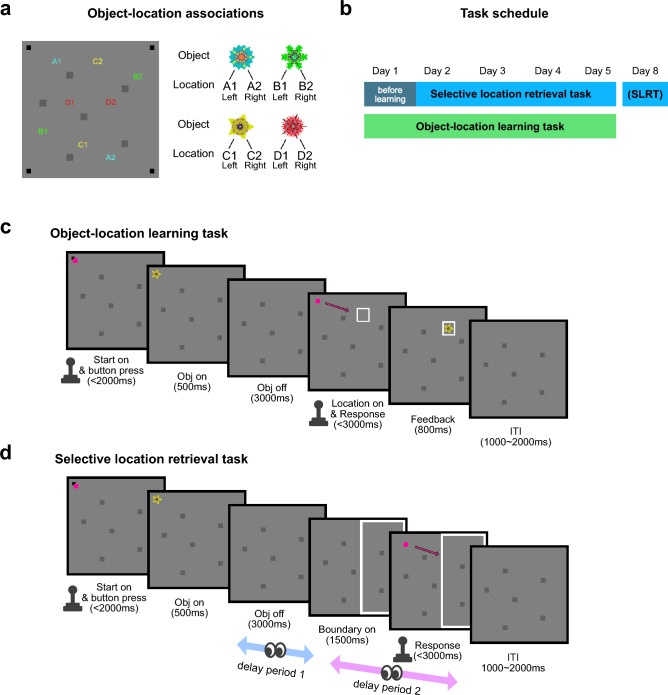
Behavioral scheme for learning of the object locations and retrieval of the learned locations. (**a**) Locations associated with each object are indicated on the background screen. The six dark grey squares were displayed as a background to provide spatial reference points. The black dots positioned at the four corners of the screen indicate the starting point locations. (**b**) The timeline of the behavioral task scheme. The selective location retrieval task was conducted before the object-location learning task. (**c**) The task design of the object-location learning task. (**d**) The task design of the selective location retrieval task (SLRT). Notably, delay periods 1 and 2 were utilized to examine the relationship between the gaze patterns and memory retrieval.

We designed two behavioral tasks in which participants learned multiple locations associated with individual fractal objects and retrieved these learned locations on next day or several days after learning the locations (Fig. 1). Specifically, each of the four visual fractal objects was associated with two locations, one located on the left side and the other on the right side of the monitor screen (Fig. 1a). The participants learned the object locations through the object-location learning task for five consecutive days (Fig. 1b and c). In the object-location learning task (Fig. 1c), a visual object and a white target box indicating one of the two associated locations were pseudorandomly presented in each trial. Participants were instructed to respond by moving the joystick to the indicated location and pressing a button, and to remember the associations between the objects and the locations.

Their memory of the object locations was tested on subsequent days using the selective location retrieval task (SLRT) (Fig. 1b and d). The SLRT on day 1 was conducted before the first object-location learning task and was included as a control task. In the SLRT, participants were presented with a learned fractal object in one of the four corners and were instructed to identify the location associated with that object inside the contextual boundary box using the joystick (Fig. 1d). Notably, to investigate the eye movements corresponding to the multi-memory retrieval, a 3-s delay period was introduced after the fractal object disappeared (delay period 1 in Fig. 1d). Subsequently, a contextual boundary box was presented the screen, and a 1.5-s period followed. This 1.5-s period and the period until the joystick response was defined as delay period 2 to examine eye movements related to context-appropriate memory retrieval (Fig. 1d). Participants were free to move their eyes throughout our tasks. No specific instructions or requirements were provided for eye movements, ensuring the examination of spontaneous eye movements during the memory retrieval processes. Furthermore, the eye movements were independent of the explicit response modality, as all responses were made with joystick manipulation in both learning and retrieval tasks.

**Figure 2:**
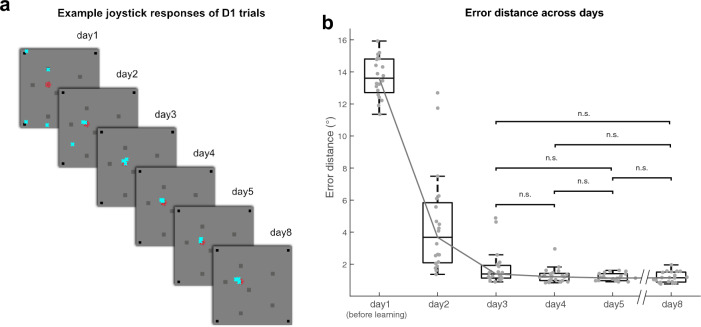
Learning of object locations over days. (**a**) Example joystick responses of a participant in the selective location retrieval task (SLRT) over the retrieval days. The cyan crosses (‘x’) indicate the participant’s response of the four trials with object D1 for each day. The location of the target object (D1 location and red fractal object) is displayed on the screen. (**b**) Boxplot diagram of the error distance of the joystick responses in the SLRT. The error distance represents the Euclidian distance between the joystick response location and the corresponding answer location for each trial. Each individual dot on the plot indicates the average error distance of all trials for a participant (n = 20). *n.s*. not significant.

In the SLRT, we confirmed that participants successfully acquired and maintained their memories of the specific locations associated with individual fractal objects over eight days (Fig. 2). Response accuracy was quantified by calculating the error distance, the spatial distance between the joystick response point and the accurate answer location. The error distance before the learning of the object locations on day 1 represents the baseline, with the joystick responses randomly distributed inside the boundary box, resulting in an error distance of 13.6036 ± 0.2708 ˚ visual angle (median ± s.e.m.) from the center of the answer locations (Fig. 2a and b). A decrease in the error distance in the SLRT indicates progressive learning of the object locations, as demonstrated by repeated measures ANOVA with retrieval days as a factor (Fig. 2b). The retrieval days had a significant main effect (F(1.5353, 29.171) = 230.8757, *p* = 2.1547 × 10^–17^, n = 20). After two days of learning, the error distance converged, indicating that participants could successfully select an object-associated location indicated by the contextual boundary box (1.3890 ± 0.2423°; median ± s.e.m. away from the center of the correct location on day 3). The error distances significantly decreased only until day 3 and showed non-significant differences from day 3 (Post-hoc Bonferroni pairwise comparisons. day 1 vs. day 2–8: *p*s ≤ 7.7163 × 10–9, day 2 vs. day 3–8: *p*s ≤ 0.0096, day 3 vs. day 4 – 8: *p*s ≥ 0.3188, day 4 vs. day 5, day 8: *p* = 1, day 5 vs. day 8: *p* = 1). As learning progressed over the days, the variability between the error distances from each correct location decreased for every fractal object (Supplementary Fig. S1). These results indicate that the participants could successfully remember the multiple locations associated with each fractal object and select the context-appropriate memory.

To further examine whether the memory of multiple locations was retained, the location memories learned for five days were tested after a three-day retention period (Fig. 1b). The results on day 8 show that participants selected the correct locations associated with each fractal object, with an average error distance of 1.1511 ± 0.0727° (median ± s.e.m.) away from the center of the correct location (Fig. 2b). These findings suggest that the memory of multiple locations associated with each fractal object was retained as a long-term memory even after a three-day retention period. Additional analysis of errors categorized into different error types of precision errors, swap errors, or random guesses also evidently show that learning converged, and the participants retained their long-term memory (Supplementary Fig. S2).

### Gaze time patterns reflect the retrieval of locations associated with objects

To investigate the relationship between eye movements and the retrieval of location memories associated with objects, we analyzed the eye movements of the participants during delay periods 1 and 2 in the SLRT (Fig. 1d). We focused on these delay periods because we assumed that the participants would retrieve multiple memories associated with the visual object during delay period 1, while they would selectively retrieve the memory relevant to context during delay period 2. We hypothesized that if the participants’ eye movements were indicative of the retrieval of object-associated location memories, they would likely gaze toward one or both locations associated with the given object during the 3-s delay period 1. In delay period 2, the presentation of the boundary box indicated the target location to be retrieved among the two associated locations. Therefore, we anticipated that the participants would gaze toward the target location within the contextual boundary box during this delay period 2.

**Figure 3:**
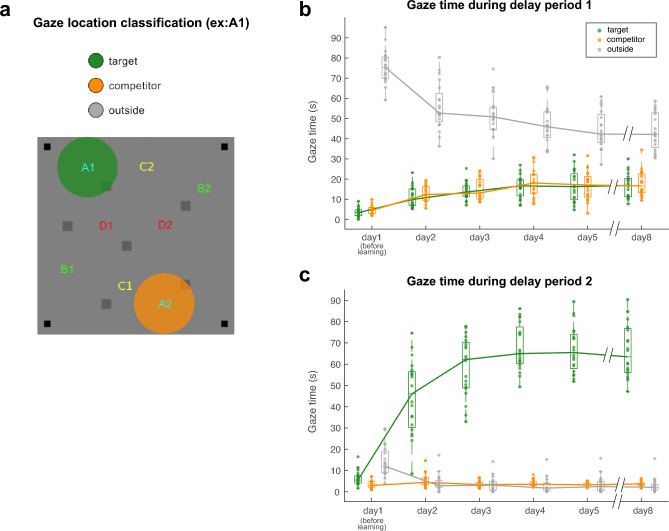
Gaze time reflects the retrieval of location memories. (**a**) Illustrative spatial windows for categorizing gaze locations. The green and orange circles represent the target (for this example: A1) and competitor windows (A2), respectively, with a radius of a 4.5° visual angle. (**b**) Boxplot diagram showing gaze time across trials by gaze location type during delay period 1 in the SLRT. Each individual dot on the plot indicates the total gaze time of all trials for each gaze location type of each participant (n = 20). (**c**) Boxplot diagram showing the gaze time across trials by gaze location type during delay period 2 in the SLRT. Each individual dot on the plot indicates the total gaze time of all trials for each gaze location type of each participant (n = 20).

To examine the presence of these eye movements related to retrieving location memories during the delay periods, we measured the duration of the participants’ gazes within 4.5° from the center of each learned object location (Fig. 3a). Two spatial windows were defined: the target object-associated location window inside the contextual boundary box and the competitor location window associated with the same object but located outside the boundary box (Fig. 3a). The 4.5° radius window was used because it ensures that the target window and the competitor location window do not overlap in each trial. All other locations outside the target and competitor locations were categorized as outside. Using this classification of gaze locations, we analyzed the duration of gaze inside each type of location (target, competitor, or outside) across the trials during each delay period.

We examined the total gaze times toward the target, competitor, and outside locations during delay period 1 of all trials in a session. A repeated-measures two-way ANOVA was conducted with retrieval days and gaze locations as factors revealing significant main effect of retrieval days (F(5, 95) = 2.4880, *p* = 0.0366) and gaze locations (F(1.0703, 20.3356) = 339.7951, *p* = 2.1956 × 10^–14^). Furthermore, this ANOVA analysis indicated a significant interaction between retrieval days and gaze locations (F(4.0304, 76.5770) = 51.1942, *p* = 5.6255 × 10^–21^). Post-hoc Bonferroni pairwise comparisons revealed that the total gaze time at both the target and competitor locations increased significantly as the retrieval days progressed (day 1 vs. day 2–8: *p*s ≤ 1.89 × 10^–4^, day 2 vs. day 4-8: *p*s ≤ 0.01 for target, day 1 vs. day 2–8: *p*s ≤ 4.85 × 10^–5^, day 2 vs day 4,8: *p*s ≤ 0.016 for competitor) (Fig. 3b and Supplementary Table S1). In contrast, the total gaze time spent outside the target and competitor locations decreased as the retrieval days progressed (post-hoc Bonferroni pairwise comparisons. day 1 vs. day 2 ~ 8: *p*s ≤ 5.87 × 10^–5^, day 2 vs. 4-8: *p*s ≤ 0.01, day 3 vs. 4,8: *p*s ≤ 0.03) (Fig. 3b and Supplementary Table S1). These results indicate that as learning occurred, participants gazed increasingly at the task-relevant, object-associated locations and decreased their gaze toward the task-irrelevant outside location during delay period 1.

During delay period 2, when the contextual boundary box was presented, we also found that the retrieval days and gaze locations had significant main effects and a significant interaction between them (repeated-measures two-way ANOVA, with days and gaze locations as factors (F(3.0194, 57.3694) = 84.4362, p = 4.4480 × 10^–21^ for days effect, F(1.1570, 21.9837) = , *p* = 1.9548 × 10^–17^ for gaze location effects, F(3.3086, 62.8640) = 104.5839, *p* = 2.6664 × 10^–25^ for days × gaze locations). Notably, there was an increase in total gaze time toward the target locations over the retrieval days (post-hoc Bonferroni pairwise comparisons. day 1 vs. day 2–8: *p*s ≤ 2.63 × 10^–7^, day 2 vs. 3–8: *p*s ≤ 0.04, day 3 vs. 4: *p* = 0.023) (Fig. 3c and Supplementary Table S3). Moreover, the total gaze time toward the target locations was significantly higher than the gaze time toward competitor and outside locations (post-hoc Bonferroni pairwise comparisons. target vs. competitor and target vs. outside on day 2–8: *p*s ≤ 5.32 × 10^–8^) (Fig. 3c and Supplementary Table S4). The gaze time toward outside and competitor locations did not differ from day 2 (*p*s ≥ 0.3848). Overall, our gaze data imply that participants recalled two locations associated with an object upon the presentation of the fractal object and later chose the memory of the target location following the introduction of the contextual boundary. Collectively, these findings indicate that gazes reflect the retrieval and selection of object-location memory.

### Participants frequently gazed at both object-associated locations during retrieval as learning occurred

**Figure 4:**
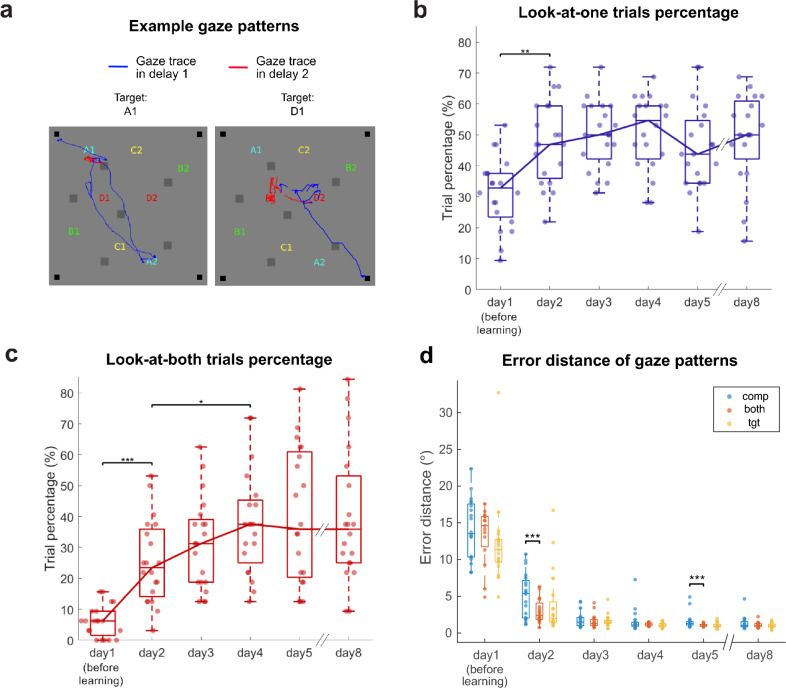
Change of gaze patterns as learning progressed and their correlation with memory accuracy. (**a**) Two example gaze patterns during retrieval phases (delay periods 1 and 2) in the SLRT. The blue and red traces represent the eye positions during delay period 1 and delay period 2, respectively. (**b**) Boxplot showing the percentage of look-at-one trials during delay period 1. (**c**) Boxplot showing the percentage of the look-at-both trials during delay period 1. (**d**) Boxplot depicting the error distance categorized by the different gaze patterns in the trials. Each individual dot represents the mean error distance of each participant in the corresponding gazing pattern trials (n = 20). Blue and red colors indicate the look-at-competitor (comp) and look-at-both trials (both), respectively. Yellow color represents the look-at-target trials (tgt). *p < 0.05, ***p < 0.001.

The example eye trace data in Fig. 4a demonstrates the gaze pattern that reflects the multi-memory retrieval and selection process during delay period 1 of a single trial. The participant alternated their gaze between the locations associated with the previously learned fractal object on the background screen (blue lines in Fig. 4a). However, after the presentation of the contextual boundary, the gaze shifted to the contextually relevant target location during delay period 2 (red lines in Fig. 4a). Analyzing the frequency of the gaze toward both remembered locations may provide insights into how often participants recalled these two locations before context presentation.

We investigated the proportion of trials in which participants gazed toward either one or both of the two locations associated with a given fractal object in each trial during delay period 1. Trials were categorized as “look-at-one” when participants gazed toward only one of the object-associated locations (either target or competitor locations, as defined in Fig. 3a), and as “look-at-both” when they alternated between both object-associated locations. The proportions of look-at-one and look-at-both trials were calculated during delay period 1 (Fig. 4b and c, respectively).

The proportion of look-at-one trials increased and remained stable after the initial day of learning (Fig. 4b). The proportion of look-at-both trials started at 5.15 ± 0.24% (mean ± s.e.m.) before object-location associative learning. As learning progressed over the days, this proportion showed an increasing pattern, reaching 38.59 ± 1.11% on day 5 (Repeated measures ANOVA with retrieval days as a factor. F(5, 95) = 20.0227, *p* = 1.3789 × 10^–13^; post-hoc Bonferroni pairwise comparisons. day 1 vs. day 2: *p* = 1.8861 × 10^–4^, day 2 vs. day 4: *p* = 0.0253) (Fig. 4c). After the 3-day retention period, the tendency to gaze at both locations associated with each fractal object was maintained (38.13 ± 1.06%, day 5 vs. day 8: *p* = 1) (day 8 in Fig. 4c). The emergence of look-at-both eye movements suggests that multiple memories were being retrieved before memory selection, and this multi-memory retrieval showed an increasing pattern as learning occurred and saturated.

### Participants showed more accurate retrieval of the target location in trials where they gazed at both locations associated with the object

Our data suggest a relationship between the gaze patterns during delay period 1 and the joystick response accuracy. Specifically, we hypothesized that memory retrieval performance would be worse (with longer error distance) in trials when participants only looked at competitor locations (look-at-competitor) compared to trials when they only looked at target locations (look-at-target) or when they looked at both locations (look-at-both). By analyzing this relationship between gaze patterns and response accuracy, we can further confirm whether gaze patterns reflect the multi-memory retrieval process and their utility as a behavioral index of the retrieval process.

To test this hypothesis, we first categorized the trials of the SLRT based on the gaze patterns during delay period 1 as look-at-target, look-at-competitor, or look-at-both trials and analyzed the error distances of the joystick responses for each category (Fig. 4d). To compare the error distances of the different trials based on the gaze patterns, a permutation-based pairwise comparison analysis was conducted (30,000 permutations for each pairwise comparison). Interestingly, we found that a day after the first learning (day 2) and on day 5, the error distances in the look-at-competitor trials were greater than those in the look-at-both trials (day 2 look-at-competitor vs. look-at-both: Bonferroni corrected *p* = 2.9999 × 10^–4^; day 5 look-at-competitor vs. look-at-both: Bonferroni corrected *p* = 9.9997 × 10^–5^). In addition, we found that pupil size of look-at-competitor and look-at-both trials were larger than look-at-target trials during delay period 2 (Permutation-based pairwise comparison analysis; day 4 look-at-competitor vs. look-at-target: Bonferroni corrected *p* = 0.0111; look-at-both vs. look-at-target *p* = 0.0453) (Supplementary Fig. S3). However, there were no differences in pupil size during delay period 1. Overall, the correlation between gaze patterns and memory accuracy further supports the idea that gaze patterns reveal object-location memories being retrieved and that multi-memory retrieval reflected by look-at-both eye movements may be beneficial in more accurate retrieval in the early phase of the learning process.

## Discussion

The present study shows the retrieval of multiple memories and context-dependent selection through human eye movements. We found that participants often spontaneously looked at both locations associated with the visual object based on multiple spatial memories, and that this behavior increased as learning occurred. This gaze behavior switched to looking at the relevant target location when the context was given, reflecting the selection of the appropriate memory. Interestingly, spontaneous and voluntary gaze patterns that participants displayed before context presentation were correlated with different response accuracies at different time points during the learning stage. Our data shows that the look-at-both behavior that naturally emerged and increased as learning saturated was related with higher memory accuracy, suggesting functional advantages of the behavior. Overall, our study provides new insights into naturalistic gaze behavior associated with multi-memory retrieval and selection across long-term learning dynamics and supports the role of eye movements as behavioral indices of memory selection processes.

Using eye movements as an index of retrieval selection provides several advantages. First, the spontaneity of eye movements provides a temporal advantage to other suggested behavioral indices. The spontaneity of eye movements can also enable trial-by-trial analysis, providing a more precise analysis of the neural mechanisms in the unit of individual trials when combined with neuroimaging techniques^7^. Furthermore, previous studies have demonstrated that memory effects on eye movements precede explicit responses and may even be independent of explicit recall, implying that gaze patterns can potentially reveal implicit cognitive processes^7,9,14,16^. Thus, eye movements are helpful for studies where verbal reports of the retrieval process cannot be obtained, such as in studies with non-human primates or infants^7^. Our study further demonstrates that eye movements can be utilized in studies of retrieval and selection of multiple memories.

Notably, our findings build upon previous research by examining naturalistic gaze behaviors linked to multi-memory retrieval and selection. Consistent with prior studies, our results corroborate that gaze locations can indicate which memory is being retrieved. This aligns with previous literature that suggest LAN behavior reflects the use of an “external memory”, where eye movements function as “spatial indexes” of sparse memory representations of the visual world^17–19^. From this perspective on naturally occurring gaze behaviors, we can infer that look-at-both trials were correlated with higher memory accuracy compared to look-at-competitor trials because both target and competitor locations are being retrieved from early in the delay period while only the competitor memory is being accessed in look-at-competitor trials. Employing the assumption that gaze location indicates the memory being retrieved, we can infer that competitor location is being retrieved in look-at-competitor and look-at-both trials, while only the target location is being retrieved before contextual boundary presentation in look-at-target trials. For successful selection of the appropriate memory after contextual boundary presentation, competition coming from the retrieved competitor location has to be resolved in look-at-both and look-at-competitor trials.

Previous literature has established that mnemonic interference from competing memories may be probed by increased pupil diameter^15,20^. Our additional analysis of pupil size differences suggests that there may be a higher cognitive load associated with interference resolution during look-at-both or look-at-competitor trials compared to look-at-target trials, particularly during delay period 2 (Supplementary Fig. S3). These results suggest that when participants looked at the location associated with the competitor, more cognitive control may be needed as they are required to inhibit the competitor memory during memory selection after context is given. On top of the previous RIF literature that proposed inhibitory process through repeated practice of a selected memory, our results overall corroborate this inhibitory process in memory selection in a trial-by-trial basis and that gaze patterns can indicate the memories that are being retrieved or processed.

Our results align with previous studies on eye movements and memory retrieval, showing an “expertise effect” in visual comprehension. In these studies, experts fixated more on task-relevant areas and made fewer fixations on task-irrelevant areas than non-experts^21^. Consistent with this effect, our data also revealed that participants demonstrated an increase in gaze time toward the relevant locations during both delay periods as they gained expertise in retrieving spatial memories associated with an object (Fig. 3). Gaze time toward the target and competitor increased over the retrieval days in delay period 1. In delay period 2, gaze time toward the target location increased over the retrieval days. Conversely, gaze time toward irrelevant locations decreased in delay period 1. Additionally, gaze time toward competitor locations, relevant to the task during delay period 1 but not in delay period 2, significantly decreased from delay period 1 to 2. These results demonstrate that gaze patterns during the SLRT reflect the learning process. The increasing focus on relevant locations as learning processes can be considered an information-reduction process, where participants optimize information processing by directing attention to task-relevant areas^22^. This aligns with the concept of adaptive gating of memory, a cognitive control mechanism that enables only the relevant information to enter the working memory space while excluding irrelevant information^23^.

The look-at-both behavior observed in our results suggests that multiple memories are often retrieved. An important question that follows is how exactly are multiple memories retrieved in the brain? Do multiple memories get retrieved simultaneously, or are they retrieved sequentially? Because eye fixations can only occur at one location at a time, we cannot dissociate sequential and simultaneous retrieval of multiple memories based solely on eye movement data. The exact mechanism of multiple memory retrieval is still being debated in the literature^24–26^.

One study supporting sequential retrieval has shown that when two memories were probed, visual working memory attends to one memory item at a time through alternating visuocortical responses^27^. On the other hand, there are also arguments for the simultaneous retrieval of memories. Competition between visual memories was correlated with more ambiguous neural patterns, suggesting that the memories were activated in parallel^28,29^. Future neuroimaging and electrophysiology studies are needed to clarify the temporal dynamics of multi-memory retrieval. These studies could provide valuable insights into whether multiple memories are retrieved sequentially or simultaneously, shedding light on the intricate processes involved in memory retrieval.

In summary, our gaze pattern data show that in retrieval situations where multiple locations are associated with a particular object, participants often gazed at multiple locations initially and then gazed at the context-appropriate location after context was given. These gaze behaviors may indicate the retrieval of multiple memories and the selection among them using contextual information. Additionally, we demonstrated that during the initial learning phase, gaze patterns associated with multi-memory retrieval may be advantageous in selective retrieval situations. Our findings emphasize the utility of eye movements as a valuable tool for probing the retrieval of multiple memories and the selection among them.

## Methods

### Participants

Twenty healthy adults (mean age 23.9 years; range 19–34; 13 female) participated in the experiment. All participants provided informed consent for the procedure. The experiments received approval from Seoul National University’s Institutional Review Board. All experiments were performed in accordance with the relevant guidelines and regulations.

### Stimuli

We used fractal objects for visual stimuli that were created by Fractal Geometry^30,31^. The fractals’ luminance was equalized with the SHINE (Spectrum, Histogram, and Intensity Normalization and Equalization) toolbox written with MATLAB (www.mapageweb.umontreal.ca/gosselif/shine). All fractals used in the experiment and the associated locations are shown in Fig. 1a.

### Task design

#### Object-location learning task

In the object-location learning task, participants learned two locations associated with each of four visual fractal objects. The participants underwent this task for five consecutive days. All locations associated with each fractal object are shown in Fig. 1a.

A black starting point was pseudorandomly presented in one of the four corners on the background screen to indicate the start of the task. The task background included six dark grey squares, serving as spatial reference points. The locations of the starting point were pseudorandomized to ensure that the participants memorized the allocentric location but not the specific hand movement or direction associated with the object. Upon the presentation of the starting point and the magenta cursor, participants were required to press the button on top of the joystick within 2000 ms to start the trial.

After pressing the joystick button, one of the fractal objects (size ~ 2° × 2° visual angle) was presented at the same position as the fixation point for a duration of 500 ms. Following a 3000 ms interval after the object disappeared, the magenta cursor reappeared at the starting location, and a white boundary was presented at one of the two locations to indicate the object’s location. Participants were instructed to manipulate the joystick, moving the magenta cursor inside the boundary box and pressing the button within 3000 ms.

Upon button press, the fractal object was presented inside the boundary box with auditory feedback. A correct response triggered a “beep” sound, indicating accurately responded trials, while an incorrect response triggered a “boo” sound, indicating that the button was pressed outside the boundary box.

The object-location learning task consisted of 128 trials. Each object-location pair was presented 16 times. The sequence of pairs was organized in a pseudorandomized manner.

#### Selective location retrieval task (SLRT)

In the selective location retrieval task, participants were tested on their memory of the locations associated with the objects. The position of the black starting point was pseudorandomly chosen from the four corners of the background screen. After the participant’s joystick button press, a previously learned fractal object appeared at the starting position for 500 ms. Following this, to record the gaze patterns related to retrieval, the background screen was displayed for 3000 ms (delay period 1).

After the delay period 1, a white contextual boundary box was presented for 1500 ms. This boundary of either the left or the right side of the screen indicated which of the two associated locations the participant should respond with.

After the 1.5 s period, the magenta cursor reappeared at the starting position, allowing participants to manipulate the joystick and press the button to indicate the remembered location within the 3000 ms time window. There was no feedback provided except for a “beep” sound to indicate a response. If the participant did not respond within 3000 ms, a “boo” sound played, and the trial was repeated. Participants were instructed to respond within the response period.

The selective location retrieval task comprised 32 trials. Each object-location pair was tested in eight trials, and the order of tested pairs was pseudorandomized.

### Apparatus

Eye-position data were acquired using the Oculomatic Pro 1000 eye tracker (Bio-Signal, Texas, USA) with a sampling rate of 1000 Hz. To manipulate the cursor on the screen, a 2-axis joystick was used (HF22S10 model, APEM). The data output from both the eye tracker and joystick were recorded using a data acquisition board (PCIe-6353, National Instruments, USA) interfaced through a shielded I/O connector block (SCB-68, National Instruments, USA).

Visual stimuli were presented via a 27-inch monitor (1920 × 1080 resolution, 240 Hz refresh rate). All behavioral tasks were controlled by a custom behavior-controlling system (Blip software; available at www.cocila.net/blip).

### Error type analysis

To categorize error types, we used the following criteria. If the joystick response location was within 1° radius from the answer location, we considered it as a correct response since the size of the visual object was about ~ 2 × 2°. If the distance between the response and the center of the answer location was larger than 1° (or in other words, inside a 2° diameter window from the center of the answer) but smaller than 4.5°, it was considered a ‘precision error’. If the response was within 1° radius window from other objects within the same contextual boundary (same hemi-screen), it was considered to be a ‘swap error’. Other responses were considered as ‘random guesses’.

### Statistical analysis

To evaluate the statistical significance of the effect of day and object-location associations on the error distance and the effects of day and looking patterns on the trial percentage, we conducted repeated-measures ANOVAs, followed by post-hoc Bonferroni pairwise comparisons. In all cases of repeated-measures ANOVAs, sphericity assumptions were examined using Mauchley’s test and adjusted using Greenhouse–Geisser correction if the assumptions were not met.

For the statistical analysis of the error distance according to the gaze patterns in the SLRT, we conducted a permutation-based pairwise comparison analysis. The permutation test has the advantage that it is applicable to various types of samples, without requiring an assumption of normalization or random sampling^32^. 30,000 permutations were carried out for each pairwise comparison. The Bonferroni correction was applied to manage the effects of multiple comparisons. All statistical analyses were conducted using MATLAB.

### Eye movement data analysis

Before each day of the experimental tasks, eye calibration was carried out. Participants were asked to look at various locations on the screen (typically four to five points on the screen that the experimenter manipulated; e.g. points located at 5° visual angle at the upper, lower, right, and left of the center of the screen). To further ensure the reliability of the calibrated eye signals, we carried out additional calibration trials for processing the eye movement data after data collection. Participants were asked to look at nine locations on the monitor screen two times each, which were spaced regularly at a 10° visual angle from the center of the screen. Eye signals during the calibration trials were collected and used to calculate the eye position offsets of vertical and horizontal position, and the gain to four directions (positive horizontal, negative horizontal, positive vertical, negative vertical). We plotted the calibrated data and raw data to ensure the calibration quality. The offsets and the gains were used to preprocess the eye movement data before analysis.

Eye movement and pupil size data were preprocessed before being analyzed. Eye blinks were removed using the “rmoutliers” function in MATLAB, where gaze data points or pupil size deviating more than three local scaled median outlier deviations (MAD) from the local median within the 500 ms sliding window (‘movemedian’, 500) were considered outliers. Any remaining gaze data time periods displaying velocities exceeding the threshold of 1000˚ visual angle/s in a 10 ms window were categorized as blinks and excluded from the data analysis^33,34^. Also, where pupil size was below or equal to 0 were considered as blinks, and the 30 ms preceding and 30 ms following data were removed^20^. On the remaining pupil size data, linear interpolation was performed to correct for the removed data.

Also, due to blinks or light reflections, there were cases where there was high-frequency oscillatory noise in the eye movement data. To remove this oscillatory noise, we excluded eye movement data in segments where there were more than three peaks or horizontal or vertical gaze data in a 50 ms sliding window (with 10 ms as sliding step size).

To analyze the gaze time for each type of gaze location (target and competitor locations), we used a window of 4.5° visual angle from the center of the visual object. Setting the window radius to be 4.5° ensures that the windows of each trial’s target and competitor windows do not overlap. An object was considered to be gazed at during a specific timepoint if its minimum window stay time was equal to or greater than 150 ms, and its maximum velocity did not exceed 50° visual angle/s in each 10 ms window. The categorization of look-at-one and look-at-both trials were as follows: we calculated the ratio (gaze time to target)/(gaze time to target + gaze time to competitor) × 100 for delay period 1 of each trial. If the resulting value was either 0 or 100, the trial was classified as a look-at-one trial. Otherwise, it was categorized as a look-at-both trial. If the value was 100, it was categorized as look-at-target trial, and if it was 0, it was categorized as look-at-competitor trials for comparing the error distance and the pupil sizes of the trial categories.

The pupil size reported in this study are in arbitrary units. This is due to the methodology employed by the Oculomatic Pro 1000 eye tracker, which computes the pupil size as the product of the width and height (in pixels) of the rectangle bounding the pupil, divided by the total number of pixels in the region of interest (ROI). The resulting value represents the pupil area within the ROI, normalized to a range of 0 to 1. It is important to note that because the pupil size is normalized by ROI area which can be adjusted during calibration process each day, only within-day analysis is possible.

### Supplementary Information

Supplementary Information.

## Data Availability

The data are available from the corresponding authors on reasonable request. Sharing and reuse of data require the expressed written permission of the authors, as well as clearance from the Institutional Review Boards.
