# Supplementary material for: Gaze patterns reflect the retrieval and selection of memories in a context-dependent object location retrieval task
Source: Sci Rep. 2024 Apr 24;14:9433. doi: 10.1038/s41598-024-59815-9 (PMC11043435; doi:10.1038/s41598-024-59815-9)
Supplement: Supplementary file 1 — Supplementary Information. [file 41598_2024_59815_MOESM1_ESM.pdf]

Supplementary Figure S1 Error distance of all object-location associations in the selective location retrieval task (SLRT). Error distance is the Euclidian distance from the joystick response location and the answer location of each trial. Each dot indicates one participant's average error distance of all trials.

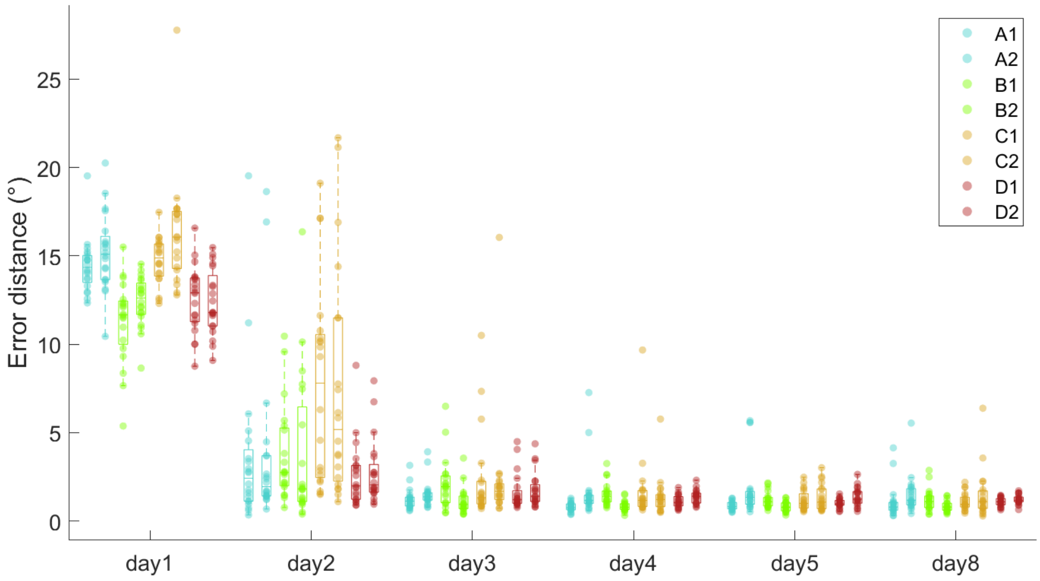

Supplementary Figure S2 Error type trial percentage in the selective location retrieval task (SLRT). The response was considered a 'precision error' if it was further than 1˚ but closer than 4.5˚ from the answer location. If it was within 1˚ radius window from other object locations within the same hemi-screen, it was considered a 'swap error. Other responses that were neither correct (within 1˚ radius window from correct location) or other errors were considered as 'random guesses'. Each dot indicates one participant's error trial percentage on each day. *p < 0.05, **p < 0.01, ***p < 0.001.

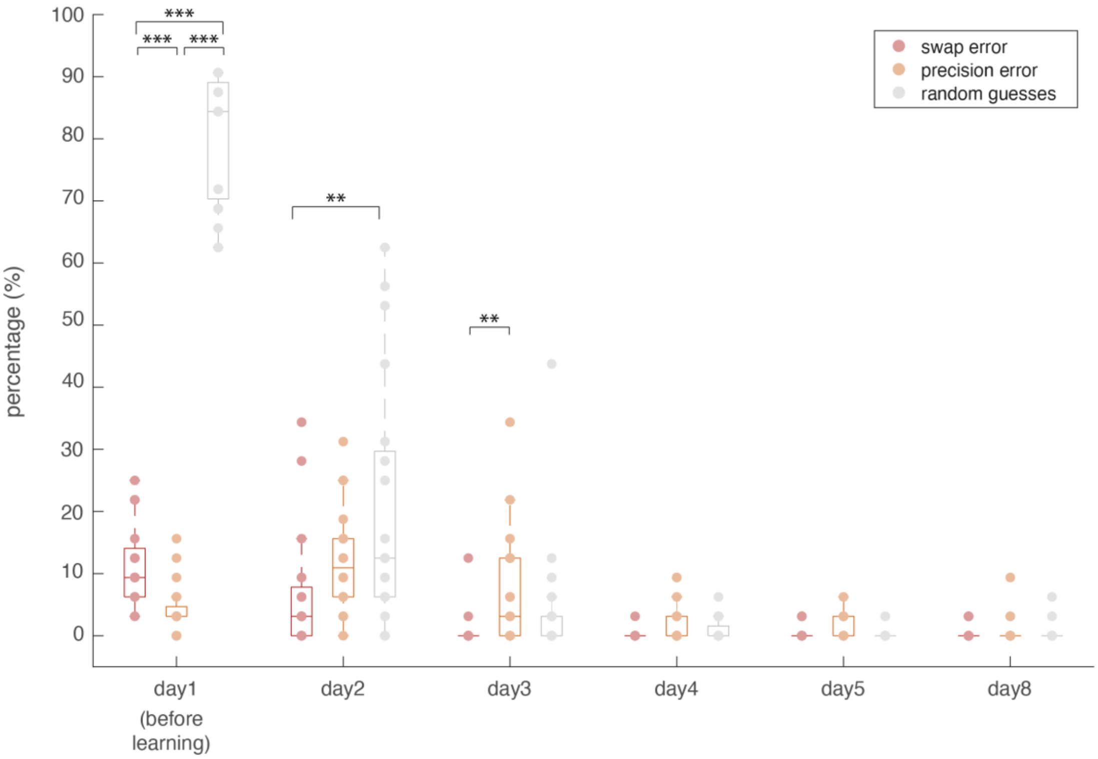

Supplementary Figure S3 Boxplot depicting the mean pupil size (arbitrary unit) of each participant in the corresponding gaze pattern trials (n = 20) in delay period 2. Blue and red colors indicate the look-at-competitor (comp) and look-at-both trials (both), respectively. Yellow color represents the look-at-target trials (tgt). *p < 0.05, **p < 0.01, ***p < 0.001.

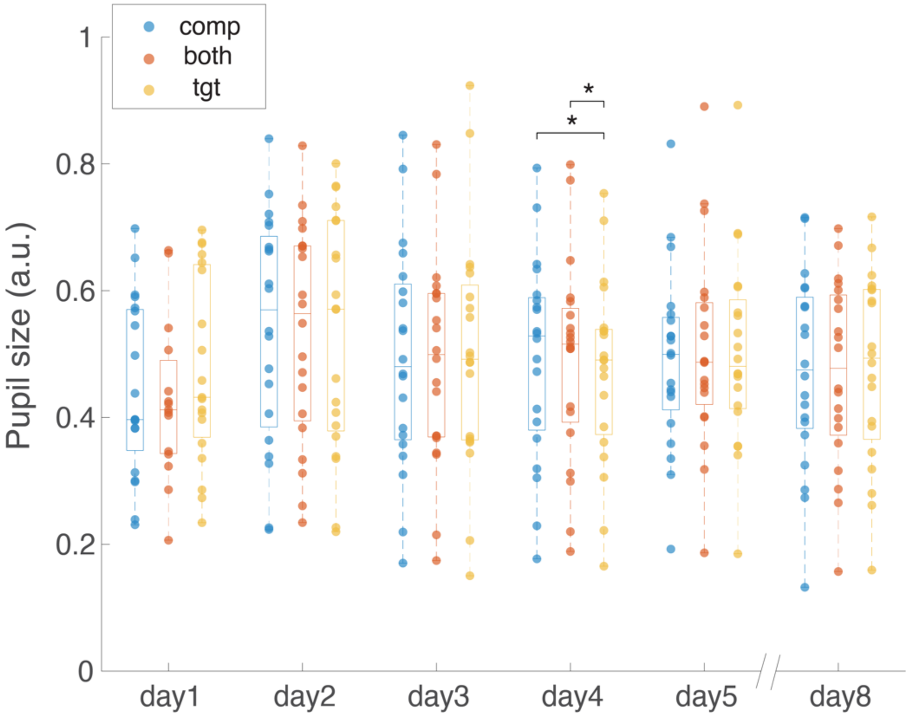

Supplementary Table S1 Delay period 1 post-hoc Bonferroni Comparisons of retrieval days

Gaze location	Day (1)	Day (2)	Difference	StdErr	pValue
Competitor	day 1	day 2	-8.0623	1.1024	9.28E-06
	day 1	day 3	-10.2183	1.4804	2.09E-05
	day 1	day 4	-13.4369	1.7911	6.44E-06
	day 1	day 5	-11.9094	1.8355	4.85E-05
	day 1	day 8	-13.1170	1.5785	1.42E-06
	day 2	day 3	-2.1561	1.1757	1
	day 2	day 4	-5.3747	1.3953	0.01612111
	day 2	day 5	-3.8472	1.5151	0.30019328
	day 2	day 8	-5.0548	1.2441	0.00995632
	day 3	day 4	-3.2186	1.3039	0.34848327
	day 3	day 5	-1.6911	1.3842	1
	day 3	day 8	-2.8987	1.4513	0.90474375
	day 4	day 5	1.5275	1.3080	1
	day 4	day 8	0.3199	1.3182	1
	day 5	day 8	-1.2076	1.1415	1
Outside	day 1	day 2	19.8284	3.1000	5.87E-05
	day 1	day 3	23.8796	2.4013	8.63E-08
	day 1	day 4	29.3814	2.1964	6.08E-10
	day 1	day 5	31.2282	2.9031	2.41E-08
	day 1	day 8	32.2236	2.7139	4.65E-09
	day 2	day 3	4.0511	2.2550	1
	day 2	day 4	9.5530	2.3529	0.01001746
	day 2	day 5	11.3998	2.5737	0.00431707
	day 2	day 8	12.3952	1.9238	5.32E-05
	day 3	day 4	5.5018	1.2884	0.00620386
	day 3	day 5	7.3487	2.4072	0.09821369
	day 3	day 8	8.3441	2.2989	0.02676521
	day 4	day 5	1.8468	1.9751	1
	day 4	day 8	2.8422	1.9741	1
	day 5	day 8	0.9954	1.5706	1
Target	day 1	day 2	-7.4490	1.2753	0.00018916
	day 1	day 3	-10.5939	1.0495	6.79E-08
	day 1	day 4	-12.6976	1.3855	3.16E-07
	day 1	day 5	-13.0219	1.8563	1.67E-05
	day 1	day 8	-13.0969	1.5014	6.79E-07

	day 2	day 3	-3.1449	1.1691	0.21751581
	day 2	day 4	-5.2486	1.1821	0.00421568
	day 2	day 5	-5.5729	1.3653	0.00953714
	day 2	day 8	-5.6479	1.3535	0.00774724
	day 3	day 4	-2.1037	0.9904	0.70510315
	day 3	day 5	-2.4281	1.6881	1
	day 3	day 8	-2.5030	1.2283	0.83599537
	day 4	day 5	-0.3243	1.3880	1
	day 4	day 8	-0.3993	1.2035	1
	day 5	day 8	-0.0750	0.9987	1

Supplementary Table S2 Delay period 1 post-hoc Bonferroni Comparisons of gaze locations

Day	Gaze location (1)	Gaze location (2)	Difference	StdErr	pValue
Day 1	Competitor	Target	1.1433	0.4582	0.06591041
	Outside	Competitor	70.9265	1.9373	1.3131E-18
	Outside	Target	72.0697	2.0446	2.6726E-18
Day 2	Competitor	Target	1.7565	0.8124	0.13072107
	Outside	Competitor	43.0358	3.0919	6.11E-11
	Outside	Target	44.7923	3.3335	1.1251E-10
Day 3	Competitor	Target	0.7677	1.1275	1
	Outside	Competitor	36.8286	3.0587	7.3408E-10
	Outside	Target	37.5963	2.7641	9.1117E-11
Day 4	Competitor	Target	1.8826	1.0857	0.29730519
	Outside	Competitor	28.1082	3.2173	1.3268E-07
	Outside	Target	29.9908	2.9437	1.1681E-08
Day 5	Competitor	Target	0.0308	1.2323	1
	Outside	Competitor	27.7889	3.3844	3.4093E-07
	Outside	Target	27.8196	3.4680	4.8294E-07
Day 8	Competitor	Target	1.1634	0.9307	0.67937356
	Outside	Competitor	25.5859	2.9592	1.5562E-07
	Outside	Target	26.7493	3.0857	1.4954E-07

Supplementary Table S3 Delay period 2 post-hoc Bonferroni Comparisons of retrieval days

Gaze location	Day (1)	Day (2)	Difference	StdErr	pValue
Competitor	day 1	day 2	-2.2624	0.9305	0.37658634
	day 1	day 3	-0.1321	0.5610	1
	day 1	day 4	-0.5620	0.5884	1
	day 1	day 5	0.1109	0.4891	1
	day 1	day 8	-0.2083	0.4464	1
	day 2	day 3	2.1303	0.8370	0.29648231
	day 2	day 4	1.7004	0.8516	0.90584581
	day 2	day 5	2.3733	0.7889	0.10842346
	day 2	day 8	2.0541	0.8740	0.44572536
	day 3	day 4	-0.4299	0.3860	1
	day 3	day 5	0.2430	0.2972	1
	day 3	day 8	-0.0762	0.4512	1
	day 4	day 5	0.6729	0.4170	1
	day 4	day 8	0.3537	0.4872	1
	day 5	day 8	-0.3192	0.3551	1
Outside	day 1	day 2	10.1278	2.1204	0.00196875
	day 1	day 3	10.7079	1.7455	0.00010134
	day 1	day 4	11.3371	1.7116	3.67E-05
	day 1	day 5	10.7547	1.7962	0.00013831
	day 1	day 8	11.3203	1.7849	6.56E-05
	day 2	day 3	0.5801	1.0010	1
	day 2	day 4	1.2093	1.0763	1
	day 2	day 5	0.6269	1.0211	1
	day 2	day 8	1.1925	1.0372	1
	day 3	day 4	0.6292	0.3467	1
	day 3	day 5	0.0468	0.4285	1
	day 3	day 8	0.6124	0.4505	1
	day 4	day 5	-0.5824	0.4710	1
	day 4	day 8	-0.0168	0.3481	1
	day 5	day 8	0.5656	0.4048	1
Target	day 1	day 2	-38.2813	4.1284	2.63E-07
	day 1	day 3	-52.4992	3.2194	1.88E-11
	day 1	day 4	-61.5615	2.4584	7.75E-15
	day 1	day 5	-60.5628	2.5316	1.80E-14
	day 1	day 8	-59.9247	2.9467	3.53E-13

	day 2	day 3	-14.2179	4.1002	0.03866902
	day 2	day 4	-23.2802	4.0879	0.00025886
	day 2	day 5	-22.2815	3.9577	0.000298
	day 2	day 8	-21.6435	4.4877	0.00177367
	day 3	day 4	-9.0623	2.4552	0.02326159
	day 3	day 5	-8.0636	3.0190	0.22656172
	day 3	day 8	-7.4255	2.9084	0.29141434
	day 4	day 5	0.9987	1.8262	1
	day 4	day 8	1.6368	1.7658	1
	day 5	day 8	0.6381	1.7819	1

Supplementary Table S4 Delay period 2 post-hoc Bonferroni Comparisons of gaze locations

Day	Gaze location (1)	Gaze location (2)	Difference	StdErr	pValue
Day 1	Competitor	Target	-2.9521	0.7848	0.00396396
	Outside	Competitor	10.7545	1.6695	1.07E-05
	Outside	Target	7.8024	2.0805	0.00406583
Day 2	Competitor	Target	-38.9710	4.2062	5.32E-08
	Outside	Competitor	-1.6357	1.0286	0.38489278
	Outside	Target	-40.6067	4.1643	2.37E-08
Day 3	Competitor	Target	-55.3191	3.1144	8.16E-13
	Outside	Competitor	-0.0856	0.8398	1
	Outside	Target	-55.4047	3.4335	4.54E-12
Day 4	Competitor	Target	-63.9515	2.4676	8.21E-16
	Outside	Competitor	-1.1447	0.9855	0.77952826
	Outside	Target	-65.0962	2.8322	7.52E-15
Day 5	Competitor	Target	-63.6257	2.4857	1.03E-15
	Outside	Competitor	0.1107	0.9679	1
	Outside	Target	-63.5151	3.0393	4.29E-14
Day 8	Competitor	Target	-62.6685	2.8678	1.90E-14
	Outside	Competitor	-0.7741	0.8552	1
	Outside	Target	-63.4426	3.2712	1.67E-13
